# Medical Practitioners and Hospital Abuse

**Published:** 1904-11-12

**Authors:** 


### Medical Practitioners and Hospital Abuse.

The provision of ready facilities whereby every member of the community, howsoever poor or humble, may obtain immediate medical advice and treatment in his hour of need is not merely a reflection of modern humanity, it is a work of the highest national importance. While few if any will deny this, it seems to be scarcely recognised by the general public that they are indebted for its realisation to-day to the noble traditions of the medical profession and to the ready and free gift at all hours of personal service by its individual members. How this fact has come to be overlooked it is not easy to explain. Were it recognised, the medical profession could reasonably hope for some gratitude in return. Whereas, now, the practitioner of medicine is compelled under heavy penalties to perform many and varied public services, some of which are extremely unpleasant, and for which he receives either a mere pittance or no reward at all; while the hospitals, under the guise of charity, are yearly robbing him of his patients in increasing numbers. At first sight, perhaps, a proposal to supply free food to those who were poor, would be highly attractive. But deeper meditation soon reveals the ruin that such a course would bring. It would convert the lazy, the degenerate, the incompetent into mere parasites; and a very superficial knowledge of zoology is sufficient to bring home the hideous forms of degeneration which are the rapid and inevitable results of such an existence. The struggle of life is as necessary for individuals as it is for the race. Let us offer a helping hand to those who are being trodden under in the struggle by all means, but to go further is to bring about an end altogether inhuman and disastrous.

Sir William Chance recently pointed out at Cardiff, in dealing with the problem of poor-law relief, that the policy of making outdoor relief the rule and indoor relief the exception inevitably leads to much abuse. The country can have as many paupers as it chooses to pay for. The same reasoning may be applied to our hospitals; they, too, can have as many out-patients as they choose. The numbers yearly applying for treatment in the out-patient departments are increasing at an ominous rate. Can it be necessary or desirable to provide upwards of 2,000,000 of the inhabitants of London with free medical treatment in one year? Would it not be a wiser policy to restrict very greatly the amount of outdoor medical relief, and with the thousands of pounds annually saved in this way, to have our in-patient accommodation the best that the world can show? In spite of the employment of almoners and investigation officers to inquire into the pecuniary position of patients, there is still great abuse, even at the hospitals which employ these checks. Indeed, the inquiry method has proved expensive and cumbersome; and although some good has been done, abuse still flourishes. Is there any other method which is worthy of trial? We have already suggested that the medical profession are entitled to some consideration from the public. Why not leave it in their hands to check hospital abuse? They would benefit by not losing patients who could afford to make payment, the hospitals would benefit by the relief of their congested and over-burdened out-patient departments, and the scheme would cost not a farthing.

In Battersea, where provident dispensaries flourish, and where the working man has learned to be proud of the local hospital, and to support it as he should, such a system has been in force for some years. The principles which govern out-patient relief at the Bolingbroke Hospital are as follows:—Any emergency case which is brought to the hospital is treated there, and if necessary is taken into the wards. If able to return home the patient is given a card which must be signed by a medical man before any further hospital treatment is given. In cases which are not of the nature of emergencies no treatment is given unless the patient has been recommended to attend the hospital by a doctor. The result is a gain in every direction. The patients learn to value the treatment they receive; the medical staff do not have their time taken up with crowds of patients suffering from chilblains, corns, and such-like minor ailments, which, more often than not, are mere excuses for a morning's gossip in the out-patient waiting-room; the medical men practising in the district regard the hospital as a friend and not as a subsidised enemy, and last, but not least, charitable subscriptions are not squandered on the unworthy, morevoer, the system entails no additional expense. The ethics observed by the local practitioners forbid any charge being made for signing a recommendation to the hospital. So far the plan has worked well, and has resulted in a great reduction of hospital abuse. It would be to the interests of the voluntary hospitals if a system of such promise were to receive a trial in other districts than Battersea.

